# Associations of adverse childhood experiences with common psychiatric disorder in later life: results from the China mental health survey

**DOI:** 10.1186/s12877-023-04421-z

**Published:** 2023-10-31

**Authors:** Jinhao Li, Zhaorui Liu, Minghui Li, Yueqin Huang, Huifang Yin, Guangming Xu, Lingjiang Li, Tingting Zhang, Jie Yan, Yaqin Yu, Xiufeng Xu, Zhizhong Wang, Yifeng Xu, Tao Li, Xiaofei Hou, Xiangdong Xu, Limin Wang, Yongping Yan, Shuiyuan Xiao, Xiangdong Du, Guohua Li

**Affiliations:** 1 Tianjin Anding Hospital, Mental Health Center of Tianjin Medical University, Tianjin, China; 2 National Clinical Research Center for Mental Disorders & Key Laboratory of Mental Health, Institute of Mental Health), Peking University Sixth Hospital, Ministry of Health (Peking University), Beijing, China; 3 Mental Health Institute, the Second Xiangya Hospital, Central-south University, Changsha, 410011 Hunan China; 4 Institute of Social Science Survey, Peking University, Beijing, 100871 China; 5 Department of Epidemiology and Biostatistics, School of Public Health, Jilin University, Changchun, China; 6 Department of Psychiatry, The First Affiliated Hospital of Kunming Medical University, Kunming, Yunnan, 650032 China; 7 Department of Epidemiology and Statistics, School of Public Health and Management, Ningxia Medical University, Yinchuan, Ningxia, 750004 China; 8 Shanghai Key Laboratory of Psychotic Disorders, Shanghai Mental Health Center, Shanghai JiaoTong University School of Medicine, Shanghai, China; 9 Mental Health Centre of West China Hospital, Sichuan University, Chengdu, 610041 Sichuan China; 10 Department of Psychiatry, Hangzhou Seventh People’s Hospital, Hangzhou, 310013 Zhejiang China; 11 The Fourth People’s Hospital in Urumqi, Urumqi, 830002 China; 12 National Center for Chronic and Non-communicable Disease Control and Prevention, Chinese Center for Disease Control and Prevention, Beijing, 100050 China; 13 Department of Epidemiology, the Fourth Military Medical University, Xi’an, 710032 Shaanxi Province China; 14 Department of Social Medicine and Health Management, School of Public Health, Central South University, Changsha, 410078 Hunan China; 15 Suzhou Guangji Hospital, Suzhou, 215008 Jiangsu China; 16 Chifeng Anding Hospital, Chifeng, 024000 Inner Mongolia Autonomous Region China

**Keywords:** Adverse childhood experiences, Psychiatric disorder, Mental health, Older adults

## Abstract

**Background:**

Associations between adverse childhood experiences (ACEs) and common psychiatric disorders among older Chinese individuals have not been well reported. The objectives of this study are to examine the prevalence of ACEs and the associations of ACEs with common psychiatric disorders among older adults in China.

**Methods:**

The study used data from the China Mental Health Survey (CMHS), a nationally representative epidemiological survey, which used computer-assisted personal interviewing (CAPI), logistic regression models were used to examine community-based adult psychiatric disorders and associated risk factors. Finally, 2,317 individuals aged 60 years or over were included in the CMHS. The national prevalence of ACEs in older adults were estimated and logistic regression were used to analyse the association between ACEs and past-year psychiatric disorders.

**Results:**

Prevalence of ACEs among older adults in China was 18.1%. The three most common types of ACEs were neglect (11.6%), domestic violence (9.2%), and parental loss (9.1%). This study proved the association between ACEs and common past-year psychiatric disorders in older adults. ACEs increased the risk of past-year psychiatric disorders in older adults. After adjustment for age, sex, marital status, employment status, education, rural or urban residence, region, and physical diseases, the association between ACEs and past-year psychiatric disorders were still significant.

**Conclusions:**

ACEs are linked to an increased risk for past-year psychiatric disorders in older adults. ACEs may have long-term effects on older adults’ mental well-being. Preventing ACEs may help reduce possible adverse health outcomes in later life.

**Supplementary Information:**

The online version contains supplementary material available at 10.1186/s12877-023-04421-z.

## Introduction

The prevalence of psychiatric disorders is about 15% among older adults aged 60 years and over [1], with mood disorder, anxiety disorder, and substance use disorder being the most common ones [2]. Many epidemiological studies have identified risk factors and protective factors for psychiatric disorders in older adults. For example, the complex interplay of chronic physical illness, gender, poor health status, loneliness, and threatening or traumatic events can be referred to as risk factors for mental disorders [3–8]. On the other hand, higher socioeconomic status, participation in social activities or sports, and a healthy lifestyle are often considered protective factors [9, 10].

ACEs, also known as childhood adversity, are stressful or traumatic events that occur during childhood (usually from birth to age 18) [11–13]. Previous studies have shown that ACEs can negatively affect individuals and can also be detrimental to a variety of health outcomes [14, 15]. The relationship between ACEs and psychopathology have been reported by researchers [16–18]. Studies have observed that people who experience ACEs are at an increased risk of developing mental health problems in later life [16–18]. At the same time, ACE-induced illnesses impose a significant health and economic burden on individuals and society [16–18]. Study in Europe and North America has found that illnesses caused by ACEs impose a significant health and economic burden on individuals and society, leading a total of 37.5 million disability-adjusted life years and $1.3 trillion annually [14].

The relationship between ACEs and mental health problems in older adults have also received widespread attention. In particular, recent studies have shown that exposure to ACEs are linked to the risk of depression, cognitive impairment, functional capability, chronic diseases, and other mental health conditions in older adults. [6, 19–26]. Despite the extensive evidence linking ACEs to multiple adverse outcomes in older adults, there are some limitations. First, previous nationally representative surveys in China reported the relationship between ACEs and depressive symptoms, cognitive function, functional capability, and chronic diseases in middle-aged and older adults. Evidence for common psychiatric disorders (e.g., mood disorder, anxiety disorder, and substance use disorder) is limited. From a life-course perspective, the persistent effects of ACEs on common psychiatric disorders have not been sufficiently described for older adults in China. Second, the definition of psychiatric disorders was not based on clinical diagnoses but instead relied on self-reported depressive symptoms or other screening questionnaires.

To our knowledge, this is the first study to investigate the prevalence of ACEs and the relationship between ACEs and past-year common psychiatric disorders in people aged 60 years or older in China. To address the potential limitations of previous studies, we explored the following questions in this study: (1) What is the prevalence of ACEs in older adults? (2) Are there associations between ACEs and past-year common psychiatric disorders in older adults?

## Methods

### Sample and procedures

Data were obtained from the China Mental Health Survey (CMHS). CMHS is the first nationally representative survey of community-based adult mental disorders and mental health services conducted in China from July 2013 to March 2015 using a face-to-face computer-assisted personal interview (CAPI). A multistage stratified random sample was used to select participants from permanent residents aged 18 years and older in 31 provinces, autonomous regions, and municipalities (excluding Hong Kong, Macau, and Taiwan). The detailed study design for the CMHS has been published [27–29].

Totally 28, 140 community adults aged 18 years and over completed Part I of the interview, which included demographic information collection and psychiatric diagnostic assessments. 9,365 respondents aged 18 years and over finished Part II interview, including assessments of pharmaco-epidemiological information and other risk factors such as childhood adversities, social networks, and family burdens. In this study, 2,317 older respondents aged 60 years or over who completed both parts of the interview were selected to enter the analysis.

The Institute of Social Science Survey (ISSS) of Peking University administered and implemented the survey. Interviewers were required to complete a seven-day interview training course and pass a test before officially starting to join the study. Various forms of quality control were implemented for this study, including data verification, audio verification, telephone verification, and field verification [27–29].

### Childhood adversities assessment

ACEs questions are included in the childhood section of Part II in the CMHS, which assessed respondents’ experience of ACEs before the age of 18. Six ACEs were extracted from 14 items in the CMHS data set, including five conventional ACEs (domestic violence, neglect, guardians with substance abuse, guardians with mental illness, and guardians with criminal behaviour ), and one ACE from the modified set (parental loss) [12]. For domestic violence and neglect, participants were asked to rate the frequency of such events on a 4-point scale (frequently, sometimes, rarely, never). Response ‘frequently’ or ‘sometimes’ were classified as having such ACE and response ‘rarely’ or ‘never’ were defined as not having such ACE. For guardians with substance abuse, guardians with mental illness and guardians with criminal behaviour, participants were also asked whether they had experienced guardians with substance abuse, mental illness and criminal behaviour (yes or no). For parental loss, participants were asked using the question “Who were the primary male and female guardians for you before 18 years old? If the answer involved ‘adoptive parents’, ‘step-parents’, ‘foster parents’, ‘other male and female relatives’, ‘male and female nannies’, ‘lack of male or female guardians’, and ‘other’, parental loss existed for the participant. The method to define ACEs was referred to previous studies [12, 25, 30]. The detailed questionnaire items and definitions of each ACE indicator are available in eTable 1 in the Supplement.

### Diagnostic assessment of Psychiatric disorders

Psychiatric disorders were diagnosed using the Chinese version of the Composite International Diagnostic Interview-3.0 (CIDI), a fully structured interview instrument administered by trained interviewers. The validity and reliability of CIDI in China have been well tested [31, 32]. The lifetime and 12-month diagnoses were made for mood disorder (bipolar I and II disorders, dysthymia, major depressive disorder, unspecified depressive disorder), anxiety disorder (panic disorder, agoraphobia, specific phobia, social phobia, obsessive–compulsive disorder, generalized anxiety disorder, posttraumatic stress disorder, unspecified anxiety disorder) and substance use disorder (alcohol abuse and dependence, drug abuse and dependence) according to the criteria and definition of the Diagnostic and Statistical Manual of Mental Disorders, Fourth Edition (DSM-IV). More detailed information about the CIDI is shown in previous study [29].

### Demographic characteristics for participants

Demographics about the participants were derived from the CMHS, including age (60–69, 70–79, 80 years and above), gender (female or male), area of residence (urban or rural), region (eastern, central, western), educational level (literate or below primary school, primary school, junior high school, senior high school and above), employment status (employment, retired, other), and marital status (married, never married, separated, widowed, or divorced). Information on chronic physical diseases was collected based on self-reports, including heart disease, high blood pressure, asthma, chronic lung disease, tuberculosis, diabetes, stroke, stomach ulcers or intestinal ulcers, rheumatic fever or arthritis, and chronic headaches. Three categories were defined by this information, including no physical disease, one or two physical diseases, and three physical diseases and over.

### Statistical analyses

The weighted methods have been published elsewhere [27]. The prevalence of ACEs and their subtypes in Chinese older people were described. Frequencies and percentages were calculated for categorical variables. Logistic regression models were established to assess the associations between different amounts of ACEs and mood disorder, anxiety disorder, and substance use disorder. Model 1 was a crude model. Model 2 and Model 3 were adjusted for age, sex, marital status, employment status, education, rural or urban residence, region, and physical diseases. All analyses were based on weighted data. A significance level test of α = 0.05 was used throughout the analyses. Statistical analyses were performed using SAS 9.4.

## Results

### Prevalence of ACEs

A total of 2,317 participants completed the survey. The weighted prevalence of ACEs among older adults in China was 18.1% (Table 1). The three most common types of ACEs were neglect (11.6%), family violence (9.2%), and parental loss (9.1%). Among the older adults in China, the prevalence of each type of ACE ranged from 0.6 to 11.6%. Overall, 18.1% of Chinese older adults experienced at least one ACE and 3.1% experienced two or more ACEs.

**Table 1:** Prevalence of ACEs in older adults by ACE type

Type of ACEs	n	Weighted prevalence
Domestic violence	264	9.2
Neglect	330	11.6
Guardians with substance abuse	35	1.0
Guardians with mental illness	74	2.4
Guardians with criminal behaviour	15	0.6
Parental loss	172	9.1
Any ACE ^a^	434	18.1
Number of ACEs		
1	339	15.0
2 and more	95	3.1

Note: Any ACE ^a^: Respondents have experienced at least one type of domestic violence, neglect, guardians with substance abuse, guardians with mental illness, and guardians with criminal behaviour or parental loss

### **Demographic characteristics for participants**

Table 2 shows the demographic characteristics of participants aged 60 and over. In total, 49.0% of the participants were male, and 6.6% of participants had a senior high school and above educational level. Among the older adults, 57.9% were aged 60–69 and 7.1% were aged 80 and older.

**Table 2:** Demographic characteristics for participants^a^

Demographic characteristics		Count	%
Age range	60–69	1642	57.9
	70–79	554	35.0
	80+	121	7.1
Sex	Female	1202	51.0
	Male	1115	49.0
Marital status	Married	1788	81.3
	Separated/Widowed/Divorced/never married	529	18.7
Employment status	Employment	246	12.7
	Retired	517	23.4
	Other*	1554	63.9
Education	Below primary school	1145	54.2
	Primary school	591	26.0
	Junior high school	380	13.2
	Senior high school and above	201	6.6
Region	Eastern	796	37.6
	Central	818	33.9
	Western	703	28.5
Area of residence	Rural	1304	56.9
	Urban	1013	43.1
Number of physical diseases	No physical disease	793	36.7
	1–2 physical diseases	1008	42.8
	3 and more physical diseases	516	20.5
Past-year anxiety disorder	No	2068	94.0
	Yes	249	6.0
Past-year mood disorder	No	2053	94.6
	Yes	264	5.4
Past-year substance use disorder	No	2212	96.5
	Yes	105	3.5
Any past-year psychiatric disorder	No	497	12.3
	Yes	1820	87.7

Note: other*: Includes searching for work(not employed), temporarily out of work, homemaker, never worked, etc

^a^:All analyses were based on weighted data

### Associations of ACEs with past-year psychiatric disorders

Table 3 shows the association between ACEs and past-year psychiatric disorders. In Model 2, the associations between ACEs and all kinds of past-year psychiatric disorders were significant. Compared with those who did not experience any ACE, older adults who experienced 2 and more ACEs were more likely to have past-year anxiety disorders (OR = 3.385; 95%CI:1.540–7.437); who experienced 1 ACE were more likely to have past-year mood disorders (OR = 1.809; 95%CI:1.059–3.090); who experienced ACE were more likely to have substance use disorder (for 1 ACE, OR = 6.235; 95%CI:2.948–13.186; for 2 and more ACEs, OR = 7.129; 95%CI: 2.425–20.961). In model 3, the dose-response effect of ACEs on past-year anxiety disorder was observed (OR = 2.165; 95%CI:1.886–5.293). As the number of ACEs experienced increases, older adults were at increased risk of developing past-year anxiety disorder. However, the dose-response effect of ACEs on past-year mood disorder, substance use disorder and any psychiatric disorder were not significant.

**Table 3:** Logistic regression analysis of the association between ACEs and past-year psychiatric disorders^a^

	OR (95% CI) by No. of ACEs		
	0	1	2 and above
Model 1^b^			
Past-year anxiety disorder	1[Reference]^c^	1.769(0.994–3.14)	4.241(1.975–9.18)***
Past-year mood disorder	1[Reference]^c^	2.192(1.264–3.803)*	3.353(1.595–7.048)*
Past-year substance use disorder	1[Reference]^c^	6.477(2.842–14.761)***	5.289(1.891–14.793)**
Any past-year psychiatric disorder	1[Reference]^c^	3.232(2.039–5.124)***	4.101(2.035–8.266)***
Model 2^d^			
Past-year anxiety disorder	1[Reference]^c^	1.563(0.881–2.775)	3.385(1.540–7.437)**
Past-year mood disorder	1[Reference]^c^	1.809(1.059–3.090)*	1.937(0.748–5.016)
Past-year substance use disorder	1[Reference]^c^	6.235(2.948–13.186)***	7.129(2.425–20.961)***
Any past-year psychiatric disorder	1[Reference]^c^	2.931(1.855–4.631)***	3.289(1.556–6.955)**
Model 3^d^			
Past-year anxiety disorder	0.640(0.360–0.936)*	1[Reference]^e^	2.165(1.886–5.293)*
Past-year mood disorder	0.553(0.324–0.944)*	1[Reference]^e^	1.071(0.385–2.979)
Past-year substance use disorder	0.160(0.076–0.339)***	1[Reference]^e^	1.143(0.386–3.388)
Any past-year psychiatric disorder	0.341(0.216–0.539)***	1[Reference]^e^	1.122(0.499–2.523)

Note:*<0.05;**<0.01***<0.001

^a^ All analyses were based on weighted data

^b^ Model 1 was the crude model

^c^ Reference: No ACE exposure

^d^ Model 2 and Model 3 were adjusted for age, sex, marital status, employment status, education, rural or urban residence, region, and physical diseases

^e^ Reference: 1 ACE exposure

## Discussion

As far as we know, this is the first study to investigate the relationship between ACEs and past-year common psychiatric disorders in older adults over 60 years of age using a nationally representative sample in China. The current study found that nearly one-fifth of older adults reported experiencing any form of ACE, and the prevalence of different ACE subtypes ranged from 0. 6% for guardians with criminal behavior to 11. 6% for neglect. This study found the association between ACEs and common past-year psychiatric disorders in older adults. ACEs were observed to increase the risk of past-year anxiety disorder, mood disorder, substance use and any psychiatric disorder in older adults.

The overall prevalence reported (18.1%) was lower than 36.3–51.7% in previous studies conducted mainly among younger population [33, 34]. The study conducted in people aged 65 years and older of the United States showed, the prevalence of ACEs was 35.9% [25]. Possible explanations for the differences between our findings and those from other countries are the differences in geographic regions, age, and sociocultural factors of the study sample. The overall prevalence of ACEs was lower than 80.9% in China Health and Retirement Longitudinal Study (CHARLS) in China by Lin et al. [19]. This huge difference might due to fewer ACEs in the current study (6 ACEs in the current study vs. 12 ACEs in that study), different instruments (CIDI in the current study vs. questionnaires of the special survey in that study ), and different participants (60 years old and above in the current study vs. 45 years old in that study).

The current study found that the most common ACEs among Chinese older adults were neglect, family violence, and parental loss, which were lower than the average of international studies of adults [12]. In this study, the neglect rate of Chinese older adults was consistent with Japanese older adults influenced by the same Eastern culture, both at 11.6% [26, 35]. This may be due to the conservative expression of emotions by the older generation of parents who are influenced by the same Asian culture. Parents sometimes had to spend much time to work, so their children might be neglected. The frequency of family violence in this study was higher than the 3.7% in Japan, and the prevalence of parental loss was lower than the 24.0% in Japan. This may provide a preliminary frame of reference for the prevalence of ACEs among older adults in China.

The current study found an association between ACEs and common psychiatric disorders in older adults. ACEs were observed to increase the risk of past-year anxiety disorder, mood disorder, substance use disorder and any psychiatric disorder in older adults. The above results are consistent with earlier studies in the United States that showed an effect of ACEs on mental health outcomes in older population [25]. Studies have shown that ACEs often occur simultaneously and that experienced one ACE increases an individual’s chances of experiencing another ACE [36–40]. The cumulative risk hypothesis suggests that the accumulation of risk factors increases the probability of adverse health outcomes [41]. Thus, it is believed that there is a cumulative relationship between the number of adverse experiences and the likelihood of mental disorders, with more adverse experiences leading to a higher risk of negative outcomes [42–44]. The results of the present study also support the cumulative risk hypothesis that the accumulation of risk factors increases the risk of adverse outcomes [45]. The trend is most evident in past-year anxiety disorder.

According to previous studies, ACEs may increase the risk of mental illness in people through the following pathways. The close association of ACEs with mental health problems can often be explained by the interconnection between genetic predisposition, epigenetic mechanisms, stress-related hormonal systems (e.g., dysregulation of the hypothalamic-pituitary-adrenal (HPA), especially its end product cortisol), and immune parameters [46]. First, the association of ACEs with psychiatric disorders may be influenced by genetics. Second, exposure to such adversities triggers epigenetic modifications in gene expression that alter brain structure and thus predispose to psychiatric disorders. Finally, exposure to ACEs may lead to changes in the HPA and autonomic axis, which are associated with stress responses. Excessive early activation can lead to dysregulation of the stress system, abnormal peripheral and central cortisol levels, decreased immune function, and increased inflammatory markers [47]. These neurobiological changes are thought to directly or indirectly increase the risk of mental health problems [35]. Another possible explanation is that the areas of the brain associated with coping and emotional self-regulation may be developmentally impaired in people who have experienced ACEs. As the result, they are more likely to develop behavioral problems in adulthood, such as high levels of smoking, alcohol abuse, and sleep disturbances, which are all recognized factors associated with risk for mental illness [33]. Further validation of the mechanism that ACEs increase the risk of mental illness in older adults is needed.

This study has important implications. First, due to the occurrence of ACEs is common and may be a risk factor for past-year common psychiatric disorders in older adults, physicians should actively ask about ACEs when older adults with psychiatric disorders seek their physicians. It is also beneficial to identify ACEs in the treatment plan for older adults with mental illness. For example, psychotherapeutic approaches (e.g., cognitive behavioral therapy) could potentially address these disadvantages. It is by changing the irrational cognitions produced by ACEs to further improve the mental health of older adults. Second, it is necessary to prevent ACEs and to develop a lifetime public health strategy to reduce the potential associated risks of mental disorders in older adults. This is especially true when childhood experiences are perceived to have a cumulative negative impact on mental health.

### Limitations

Some limitations of the present study deserve comment. First, retrospective reporting of ACEs that occurred during childhood in this study may be influenced by recall bias or current emotional state. However, retrospective reporting is an accepted method in demographic studies [37]. Furthermore, previous studies have shown that retrospective measures are reliable and cannot be simply replaced by prospective measures [38]. In Asian societies, reporting of ACEs may be culturally influenced, and reporting incidents of neglect and family violence may be perceived as filial violations. In this study, the principle of complete confidentiality was explained in detail to the respondents and to ensure that all respondents completed the questionnaire as truthfully as possible. Second, ACEs were coded in two, where we did not consider the duration and severity of specific ACEs. Finally, even though a range of ACE indicators were included in the current study, some ACE indicators [12, 25] such as sexual abuse and economic adversity were not included.

## Conclusions

In summary, the prevalence of ACEs in Chinese older adults was 18.1%. ACEs were associated with past-year common psychiatric disorders even after controlling for other important covariates. ACEs may have long-term effects on the mental health of older adults and should be taken into consideration. Although further research is needed, prevention and early treatment of ACE may reduce the prevalence of mental disorders in older adults in the community.

### Electronic supplementary material

Below is the link to the electronic supplementary material.

Supplementary Material 1

## Data Availability

The data that support the findings of this study are available from the corresponding authors on request.

## Abbreviations

CMHS: China Mental Health Survey
CAPI: computer-assisted personal interview
ISSS: The Institute of Social Science Survey
ACEs: Adverse Childhood Experiences
HPA: hypothalamic-pituitary-adrenal
CHARLS: China Health and Retirement Longitudinal Study
