# Supplementary material for: Associations of adverse childhood experiences with common psychiatric disorder in later life: results from the China mental health survey
Source: BMC Geriatr. 2023 Oct 31;23:706. doi: 10.1186/s12877-023-04421-z (PMC10619228; doi:10.1186/s12877-023-04421-z)
Supplement: Supplementary file 1 — Supplementary Material 1 [file 12877_2023_4421_MOESM1_ESM.docx]

**Supplementary Table1**

**Each ACE indicator questionnaire item**

| **Types of ACE** | **Questionnaire Items** | **Response** | **Operationalization** |
| --- | --- | --- | --- |
| Neglect | How often parents or other guardians ask you to do work that is difficult or dangerous for a child of your age? | 1=Frequently  2=Sometimes  3=Rarely  4=Never | Response ‘1’ or ‘2’ to any of these item were defined as having neglect. Response ‘3’ or ‘4’ were defined as not having neglect. |
|  | How often were you left alone somewhere and unsupervised when you're too young ? |  |  |
|  | How often did you lack essential items such as clothes, shoes, and school supplies because your parents or guardians spend the money on themselves? |  |  |
|  | How often did your parents or other guardians let you go hungry or not prepare normal meals? |  |  |
|  | How often did your parents or guardians didn't pay attention when you were sick or injured, or didn't take you to treatment? |  |  |
| Domestic violence | When you were growing , how often did someone in your family do these things to you (push, grab, choke, hit,shove,slap,or throw things)? | 1=Frequently  2=Sometimes  3=Rarely  4=Never | Response ‘1’ or ‘2’ to any of these item were defined as having domestic violence. Response ‘3’ or ‘4’ were defined as not having domestic violence. |
|  | When you were growing, did your parents or guardians (push, grab, choke, hit,shove,slap,or throw things) each other? |  |  |
| Guardians with substance abuse | Did your male or female guardians ever have alcohol or drugs abuse? | 1=Yes  2=No | 1=yes;  0=otherwise |
| Guardians with  mental illness | Did the guardian’s unhappiness, low mood or sadness ever get in the way of her life or activities? | 1=Yes  2=No | 1=yes to any;  0=otherwise |
|  | Did mental stress ever interfere significantly with guardians’ life or activities? |  |  |
|  | Did guardians ever say that when an anxiety attack he feels heart pounds, breathe trouble,body uncomfortable, or afraid him will die? |  |  |
| Guardians with criminal behaviour | Did your male or female guardians have been involved (participated) in criminal activity, such as theft or selling stolen goods? | 1=Yes  2=No | 1=yes to any;  0=otherwise |
|  | Did the guardians have been arrested or served time in prison? |  |  |
| Parental loss | Who were the primary male and female guardians for you before 18 years old ? | 1=Birth parents  2=Adoptive parents  3=Step-parents  4=Foster parents  5=other male and female relatives  6=Male and female nannies  7=lack of male or female guardians  8=Other | Response ‘2’, ‘3’, ‘4’, ‘5’, ‘6’, ‘7’, and ‘8’ were defined as parental loss. Response ‘1’ was defined as not having parental loss. |
